# Popular Lectures on Man, His Structure and Functions, Considered with Reference to Health, Culture, and Natural Theology

**Published:** 1842-01

**Authors:** 


Art. X. — *Popular Lectures on Man, his Structure and Functions, considered with reference to Health, Culture, and Natural Theology.* Illustrated with Woodcuts. By John White, Surgeon.—*London,* 1841. 8vo, pp. 252.

This little book consists of Seven Lectures which were delivered at the Highgate Literary and Scientific Institution. The fact of lectures of the kind being in request at so small a place as Highgate is creditable to the character of its inhabitants, and the lectures themselves are very creditable to their author. They convey, in a simple form, what the title-page indicates, and will be found both pleasant and useful to teachers and to parents who desire the best kind of knowledge for their pupils and their children.

